# Exploring the genetic control of glycolytic oscillations in *Saccharomyces Cerevisiae*

**DOI:** 10.1186/1752-0509-6-108

**Published:** 2012-08-24

**Authors:** Thomas Williamson, Delali Adiamah, Jean-Marc Schwartz, Lubomira Stateva

**Affiliations:** 1 Faculty of Life Sciences, University of Manchester, Manchester, M13 9PT, UK; 2 Manchester Institute of Biotechnology, Faculty of Life Sciences, University of Manchester, 131 Princess St, Manchester, M1 7DN, UK

**Keywords:** Glycolytic oscillations, *Saccharomyces cerevisiae*, deletion mutants, cAMP-PKA signal transduction pathway

## Abstract

**Background:**

A well known example of oscillatory phenomena is the transient oscillations of glycolytic intermediates in *Saccharomyces cerevisiae,* their regulation being predominantly investigated by mathematical modeling. To our knowledge there has not been a genetic approach to elucidate the regulatory role of the different enzymes of the glycolytic pathway.

**Results:**

We report that the laboratory strain BY4743 could also be used to investigate this oscillatory phenomenon, which traditionally has been studied using *S. cerevisiae* X2180. This has enabled us to employ existing isogenic deletion mutants and dissect the roles of isoforms, or subunits of key glycolytic enzymes in glycolytic oscillations. We demonstrate that deletion of *TDH3* but not *TDH2* and *TDH1* (encoding glyceraldehyde-3-phosphate dehydrogenase: GAPDH) abolishes NADH oscillations. While deletion of each of the hexokinase (HK) encoding genes (*HXK1* and *HXK2*) leads to oscillations that are longer lasting with lower amplitude, the effect of *HXK2* deletion on the duration of the oscillations is stronger than that of *HXK1*. Most importantly our results show that the presence of beta (Pfk2) but not that of alpha subunits (Pfk1) of the hetero-octameric enzyme phosphofructokinase (PFK) is necessary to achieve these oscillations. Furthermore, we report that the cAMP-mediated PKA pathway (via some of its components responsible for feedback down-regulation) modulates the activity of glycoytic enzymes thus affecting oscillations. Deletion of both *PDE2* (encoding a high affinity cAMP-phosphodiesterase) and *IRA2* (encoding a GTPase activating protein- Ras-GAP, responsible for inactivating Ras-GTP) abolished glycolytic oscillations.

**Conclusions:**

The genetic approach to characterising the glycolytic oscillations in yeast has demonstrated differential roles of the two types of subunits of PFK, and the isoforms of GAPDH and HK. Furthermore, it has shown that *PDE2* and *IRA2*, encoding components of the cAMP pathway responsible for negative feedback regulation of PKA, are required for glycolytic oscillations, suggesting an enticing link between these cAMP pathway components and the glycolysis pathway enzymes shown to have the greatest role in glycolytic oscillation. This study suggests that a systematic genetic approach combined with mathematical modelling can advance the study of oscillatory phenomena.

## Background

**Figure 1:**
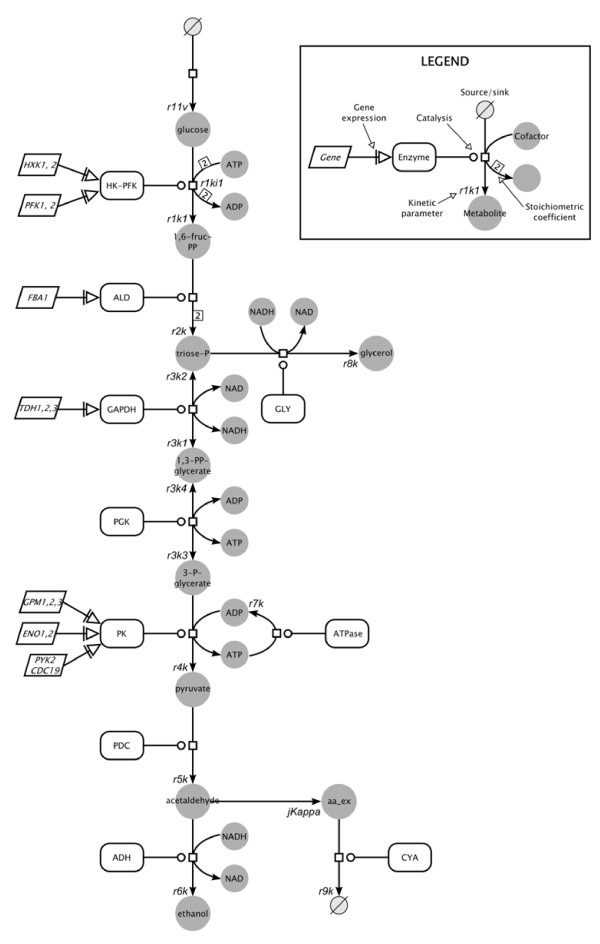
**A scheme of the glycolytic pathway in*S. cerevisiae*with respective Wolf glycolysis model parameters.** Legend inset representing respective symbols and components. Respective reactions’ abbreviations used: HK-PFK – lumped hexokinase and phosphofructokinase; ALD – aldolase; GLY – glycerol- producing branch; GAPDH – glyceraldehyde-3-phosphate dehydrogenase; PGK – phosphoglycerate kinase; PK – lumped reactions of phosphoglycerate mutase, enolase and pyruvate kinase; PDC – pyruvate decarboxylase; ADH – alcohol dehydrogenase; CYA – acetaldehyde degradation. For description of all parameters and their initial values, see Table 2.

Glucose is the major source for energy production. It is converted to pyruvate via the glycolysis pathway, leading to production of ATP coupled with generation of intermediates and reducing power in the form of NADH for other biosynthetic pathways (Figure 1). When glucose-starved *Saccharomyces cerevisiae* cells are incubated with glucose in the presence of the respiration inhibitor cyanide, transient oscillations of levels of relevant glycolytic metabolites can occur. These include levels of nicotinamide adenine dinucleotide (NAD) which can be seen to oscillate between the oxidized (NAD^+^) and reduced forms (NADH), as well as other glycolytic intermediates, including glucose-6-phosphate, fructose-6-phosphate and fructose 1,6-bisphosphate [1]. Glycolytic oscillations are accompanied by oscillations in mitochondrial membrane potential (Δψ_*m*_) [2]. This phenomenon has been observed for over 40 years [3] and is known as glycolytic oscillations [4]. These oscillations are not limited to *S. cerevisiae*, as they have been observed in *Saccharomyces carlsbergensis*[5], muscle extracts [6], and pancreatic β-cells [7]. Glycolytic oscillations are an example of oscillatory phenomena, with others including waves of cAMP in *Dictyostelium*[8], circadian rhythms [9], oscillations of Ca^2+^ levels [10] and the cell cycle [11].

Different factors have been shown to control the oscillations. These include temperature [12], cell density [13] and plasma membrane H^+^ ATPase and mitochondrial ATP-ase [14]. It is believed that either acetaldehyde or ethanol acts as a synchronization mediator [15,16] although the evidence for the role of ethanol had been disputed [15-17]. The controller of the oscillations (the so called “oscillophore”) has attracted a lot of attention but has yet to be fully established. The contributions of each element of the glycolytic pathway have been studied with mathematical models of yeast glycolytic oscillations [18-20]. Of the different glycolytic enzymes, phosphofructokinase (PFK), with its allosteric regulation (it is inhibited by ATP while AMP and fructose 1,6-bisphosphate reversing the inhibition) has been shown to have the greatest control over the oscillations. A role for the hexokinases has also been demonstrated [21]. In contrast, there is evidence that each glycolytic enzyme plays a role in controlling the oscillatory phenomenon – this is described as “distributed control” [22]. Both theories have been put forward as a result of analysis of the mathematical models that have been developed to explain the regulation of yeast glycolytic oscillations [17,20].

To our knowledge there has not been previously an attempt to investigate the yeast glycolytic oscillations at genetic level. This could be due to the fact that until now, the oscillations have only been observed in the diploid *S. cerevisiae* strain X2180. This strain has never been used to generate targeted deletion mutants; therefore, the potential effects of deletion or over-expression of glycolytic genes have, until now, remained unknown. In contrast, almost complete collections of isogenic deletion mutants are available in the BY4743 sequenced standard laboratory genetic background [23].

In this study, we have demonstrated that glycolytic oscillations can be observed in the diploid *S. cerevisiae* strain BY4743. We have subsequently used the genetic resources available in this genetic background to investigate the effects of deletion of different glycolytic enzymes encoding genes on the NADH-mediated glycolytic oscillations in the respective mutants. We have observed differential roles of the two subunit types of the phosphofructokinase, as well as the different isoforms of the hexokinase and the glyceraldehyde-3-phosphate dehydrogenase. We have used this experimental data to evaluate via parameter sensitivity analysis and representative simulations the mathematical model of yeast glycolytic oscillations developed by Wolf et al. [18]. Furthermore we have provided evidence for a role of the cAMP signal transduction pathway in modulating glycolytic oscillations.

## Methods

### Strains and media

The strains used in this study are listed in Table 1. Standard minimal (SD) with required strain-specific supplements, and rich (YPD) media, were prepared as described by Sherman et al. [24].

**Table 1:** Strains used in this study

**Strain**	**Gene deletion**	**Genotype**	**Source**
X2180	-	*MAT a/α SUC2 mal mel gal2 CUP1*	Andersen, AZ
BY4743	-	*MAT a/α; his3Δ1/his3Δ1; leu2Δ0/leu2Δ0; lys2Δ0/LYS2; MET15/met15Δ0; ura3Δ0/ura3Δ0*	Euroscarf
Y35867	*hxk1*∆	BY4743; *YFR053C::kan*MX4/*YFR053C::kan*MX4	Euroscarf
Y34620	*hxk2*∆	BY4743; *YGL253W::kan*MX4/*YGL253W::kan*MX4	Euroscarf
Y33717	*gpm2*∆	*BY4743; YDL021W::kan*MX4/*YDL021W::kan*MX4	Euroscarf
Y31748	*gpm3*∆	BY4743; *YOL056W::kan*MX4/*YOL056W::kan*MX4	Euroscarf
Y31371	*tdh1*∆	BY4743; *YJL052W::kan*MX4/*YJL052W::kan*MX4	Euroscarf
Y36806	*tdh2*∆	BY4743; *YJR009C::kan*MX4/*YJR009C::kan*MX4	Euroscarf
Y34822	*tdh3*∆	BY4743; *YGR192C::kan*MX4/*YGR192C::kan*MX4	Euroscarf
Y35893	*pfk1*∆	BY4743; *YGR240c::kan*MX4/*YGR240C::kan*MX4	Euroscarf
Y30791	*pfk2*∆	BY4743; *YMR205C::kan*MX4/*YMR205C::kan*MX4	Euroscarf
Y37286	*eno1*∆	BY4743; *YGR254W::kan*MX4/*YGR254W::kan*MX4	Euroscarf
Y31644	*pyk2*∆	BY4743; *YOR347C::kan*MX4/*YOR347C::kan*MX4	Euroscarf
Y31657	*pde2*∆	BY4743; *YOR360C::kan*MX4/*YOR360c::kan*MX4	Euroscarf
Y34615	*pde1*∆	BY4743; *YGL248W::kan*MX4/*YGL248W::kan*MX4	Euroscarf
Y31261	*tpk1*∆	BY4743; *YJL164C::kan*MX4/*YJL164C::kan*MX4	Euroscarf
Y31089	*tpk2*∆	BY4743; *YPL203W::kan*MX4/*YPL203W::kan*MX4	Euroscarf
Y35016	*tpk3*∆	BY4743; *YKL166C::kan*MX4/*YKL166C::kan*MX4	Euroscarf
Y33731	*gpr1*∆	BY4743; *YDL035C::kan*MX4/*YDL035C::kan*MX4	Euroscarf
Y30152	*gpa2*∆	BY4743; *YER020W::kan*MX4/*YER020W::kan*MX4	Euroscarf
Y31772	*ira2*∆	BY4743; *YOL081W::kan*MX4/*YOL081W::kan*MX4	Euroscarf

### Growth of strains

The strains were grown essentially as described by Poulsen et al. [25]. For each strain, a single colony was used to inoculate minimal media containing appropriate supplements, and 100 mM potassium phthalate at pH 5. The cultures were incubated at 30°C overnight with shaking, and used to inoculate 200 ml of the same synthetic media. Strains were grown at 30°C with shaking until glucose was depleted (approximately 16–20 hours). The level of glucose in the media was tested with Clinistix glucose strips (Bayer). Cells were harvested by centrifugation at 5000 rpm, washed twice with buffer (50 mM K_2_HPO_4_, pH 6.8) and suspended to 10% wet weight in the same buffer. They were then incubated at 30C with shaking for three hours and kept on ice until use.

### Measurement and induction of oscillations

Oscillations were followed using a protocol adapted from Poulsen et al. [26]. Following harvesting, 3 ml of yeast suspension was placed in a 4.5 ml PMMA cuvette (Fisher). The cuvette was placed in a Varian Carey Eclipse fluorescence spectrophotometer, and the temperature of the cell suspension was adjusted to 30°C. Cells were stirred at all times during the experiment using a magnetic stirrer. NADH fluorescence was followed with an excitation wavelength of 366 nm and an emission wavelength of 450 nm. Other settings were optimized so the measured intensity was always between 10 and 30 arbitrary units. During each run, the intensity was sampled 10 times every second. Oscillations were induced by the addition of glucose to a concentration of 30 mM after 60 seconds, followed by addition of KCN to a final concentration of 5 mM after 140 seconds. NADH levels were followed until the oscillations ceased (around 22 minutes for wild-type strains).

### Mathematical analysis

Frequency and amplitudes of oscillations were determined from Discrete Fourier Transformations (DFTs), found with Fast Fourier Transformations (FFTs) carried out in Matlab. DFTs are calculated according to the equation below:

(1) $$X\left(k\right)=\Sigma_{n=1}^{N}x\left(n\right)e^{–j2\pi \left(k-1\right)}\left(\frac{n-1}{N}\right),1\leq k\leq N-$$

This equation gives the FFT of the data. The frequency is found by taking the maximum frequency value from a plot of frequency versus power spectrum.

The steady-state parameter sensitivity analysis was carried out using SBToolbox [27] in Matlab. The SBparameterestimation function was used to calculate normalized parameter sensitivities. The SBToolbox functions SBsensamplitude and SBsensperiod were used to find the sensitivities of the amplitude and periodicity of the oscillations.

The non-normalised amplitude sensitivity for the *i*-th component of the model with respect to the *j*-th parameter is defined by:

(2) $$s_{i\;j}=\frac{A_{i}\left(p_{j}+\delta p_{j}\right)-A_{i}\left(p_{j}\right)}{\delta p_{j}}$$

where *p*_*j*_ is the reference parameter value, *δp*_*j*_ is the perturbation applied to the parameter, *A*_*i*_(*p*_*j*_) is the amplitude for the reference parameter value and *A*_*i*_(*p*_*j*_ + *δp*_*j*_) is the amplitude for the perturbed parameter value*.*

The normalised amplitude sensitivity is then defined by:

(3) $$sn_{\mathrm{ij}}=\frac{p_{j}}{Ai\left(p_{j}\right)}s_{i\;j}$$

The same definitions apply to the period sensitivity by replacing amplitudes with periods. These functions were used in this form by Stelling et al. [28].

The point in which the sensitivity analysis was carried out is defined by the reference conditions provided by Wolf et al. [18]. These include the parameters as defined in Figure 1 and Table 2, the glucose influx (*J*_0_) of 50.0 mM min^-1^, the combined pool of NAD^+^ and NADH of 1.00 mM, and the combined ADP and ATP pool of 4.00 mM. Perturbation analysis was carried out by changing the value of the relevant parameter in 1% increments for up to 10% and running the model in Matlab.

**Table 2:** Parameters and initial values of the Wolf model of glycolytic oscillations

**Parameter**	**Description**	**Initial value**
*r1k1*	Rate constant for lumped HK-PFK reaction	550 mM^-1^ · min^-1^
*r1ki1*	Inhibition constant for lumped HK-PFK reaction	1 mM
*r2k*	Rate constant for aldolase reaction	9.8 min^-1^
*r3k1*	Forward rate constant for GAPDH reaction	323.8 mM^-1^ · min^-1^
*r3k2*	Reverse rate constant for GAPDH reaction	57823.1 mM^-1^ · min^-1^
*r3k3*	Forward rate constant for PGK reaction	76411.1 mM^-1^ · min^-1^
*r3k4*	Reverse rate constant for PGK reaction	23.7 mM^-1^ · min^-1^
*r4k*	Rate constant for PK reaction	80 mM^-1^ · min^-1^
*r5k*	Forward rate constant for PDC reaction	9.7 mM^-1^ · min^-1^
*r6k*	Forward rate constant for ADH reaction	2000 mM^-1^ · min^-1^
*r7k*	Rate constant for ATPase reaction	28 min^-1^
*r8k*	Rate constant for GLY reaction	85.7 mM^-1^ · min^-1^
*r9k*	Rate constant for CYA reaction	80 min^-1^
*r11v*	Rate constant for glucose import	50 mM min^-1^
*jKappa*	Rate constant for acetaldehyde diffusion	375 min^-1^

## Results

**Figure 2:**
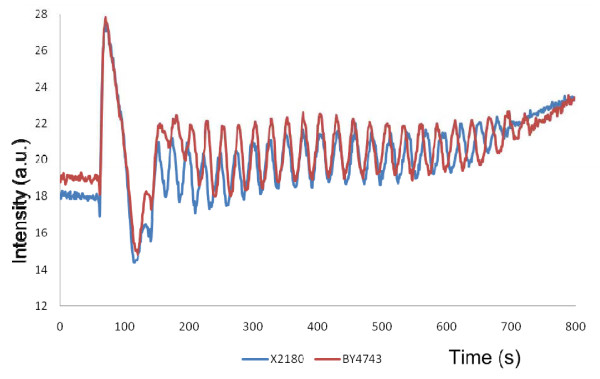
**Glycolytic oscillations in wild-type yeast strains X2180 (blue trace) and BY4743 (red trace).** Oscillations were induced by the addition of glucose to a concentration of 30 mM after 60 seconds, followed by addition of KCN to a final concentration of 5 mM after 140 seconds. NADH fluorescence intensity was followed using a fluorimeter.

**Glycolytic oscillations in S. cerevisiae BY4743 are comparable to those in X2180** Until this study, yeast glycolytic oscillations have been investigated in *S. cerevisiae* X2180, a strain rarely used in genetic analysis. We demonstrate for the first time that glycolytic oscillations could also be observed in the standard reference genetic background strain BY4743. As shown in Figure 2, the NADH-mediated oscillatory pattern of strain BY4743 is comparable to that of X2180. The conclusion is further supported by the results of a Fourier transformation analysis comparing the frequency of oscillations of the two wild type strains showing that the maximum periodicity is 26.05 seconds per cycle for BY4743 and 27.67 seconds for X2180; the difference between the two values not being significant. Both strains displayed glycolytic oscillations upon growth in minimal buffered (SD) but not rich (YPD) medium (data not shown). The use of strain BY4743 for the study of glycolytic oscillations allowed a full scale genetic investigation as isogenic homozygous diploid deletion mutants of nonessential genes encoding glycolytic enzymes are available [23].

### Respective deletion mutants reveal a role of several glycolytic enzymes in glycolytic oscillations

**Figure 3:**
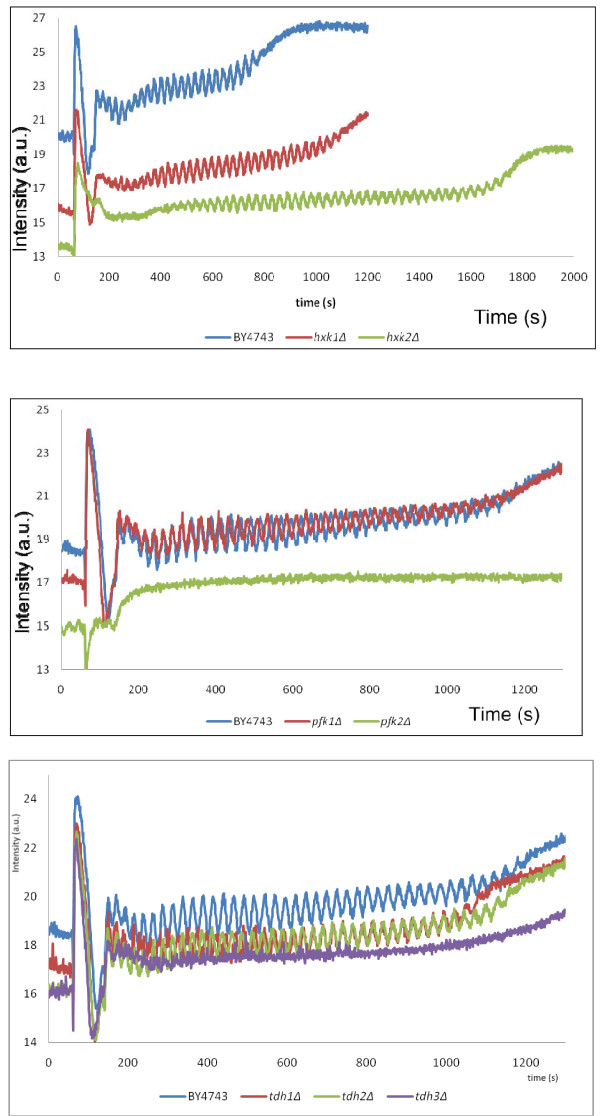
**Glycolytic oscillations in the wild type BY4743 compared to isogenic mutants in genes encoding respective glycolytic enzymes.** Panels: (**a**): BY4743 (blue trace), *hxk1Δ* (red trace) and *hxk2Δ* (green trace) mutants; (**b**): BY4743 (blue trace), *pfk1Δ* (red trace) and *pfk2Δ* (green trace); (**c**): BY4743 (blue trace), *tdh1Δ* (red trace), *tdh2Δ* (green trace) and *tdh3Δ* (purple trace). Oscillations were induced by the addition of glucose to a concentration of 30 mM after 60 seconds, followed by addition of KCN to a final concentration of 5 mM after 140 seconds. NADH fluorescence intensity was followed using a fluorimeter.

The phosphorylation of glucose, the first irreversible step of glycolysis, is catalysed by hexokinase (HK) which has two isoforms encoded by *HXK1* and *HXK2*. Our experiments showed that deletion of the hexokinase encoding genes had no effect on the frequency of the oscillations (Figure 3a). However deletion of either *HXK1* or *HXK2* decreased the amplitude of the oscillations. In contrast, while both deletion mutants sustained longer oscillations, deletion of *HXK2* had a greater effect on the duration of the oscillations than that of *HXK1.*

The phosphofructokinase has long been considered to be the key regulator of yeast glycolytic oscillations [3,29,30]. It is a complex hetero-oligomeric enzyme composed of four alpha (encoded by *PFK1*) and four beta (encoded by *PFK2*) subunits. Unlike mathematical modelling analysis which does not discriminate the two subunit types, our genetic approach was able to test their individual contributions in glycolytic oscillations. Deletion of *PFK1* resulted in a slight decrease in oscillation frequency. In contrast, deletion of *PFK2* abolished oscillations completely (Figure 3b). Therefore, while the strains that have only one of the Pfk subunits can ferment glucose [31], the homomeric nature of the PFK enzyme in these strains results in differences in the glycolytic oscillations phenotype with the *pfk2*Δ but not the *pfk1*Δ deletion mutant being affected in NADH-mediated glycolytic oscillations. Furthermore, the characteristic “spike” of NADH that we observed in all other strains was absent in the *pfk2*Δ but not in the *pfk1*Δ strain. The *pfk2*Δ mutant was the only mutant displaying a drop in NADH levels after glucose addition.

Glyceraldehyde-3-phosphate dehydrogenase (GAPDH) catalyses the reaction of glyceraldehyde-3-phosphate to 1,3 bis-phosphoglycerate. The enzyme has three isoforms encoded by three unlinked genes, *TDH1*, *TDH2*, and *TDH3*. All isoforms are catalytically active and have different specific enzymatic activities [32,33]. While Tdh2p and Tdh3p are present in exponentially growing cells Tdh1p is the predominant isoform in stationary phase [34]. As shown in Figure 3c, deletion of *TDH1* and *TDH2* had no effect on the glycolytic oscillations. In contrast, deletion of *TDH3* abolished them almost completely.

Phosphoglycerate mutase mediates the conversion of 3-phosphoglycerate to 2-phosphoglycerate during glycolysis, and the reverse reaction during gluconeogenesis. Three genes: *GPM1,2,3*, code for phosphoglycerate mutase in the yeast genome. However, while *GPM1* is functional and essential for viability [35], *GPM2* and *GPM3* are most-likely non-functional homologues of *GPM1*, since mutation in neither *GPM2* nor *GPM3* appears to affect glycolysis, or confer any obvious phenotype. In the current investigation, glycolytic oscillations in the *gpm2*Δ and *gpm3*Δ mutants were found to be wild type (data not shown). This observation gives further support to the theory that *GPM2* and *GPM3* were generated from *GPM1* by a gene duplication event and then diverged from the parent copy by mutation [36].

There are two phosphopyruvate hydratase isoforms encoded by *ENO1* and *ENO2* respectively. Both function in dimeric complexes [37] and catalyze the conversion of 2-phosphoglycerate to phosphoenolpyruvate during glycolysis. The diploid homozygous *eno1*Δ was available in the EUROSCARF collection but *eno2*Δ was not. When tested in our experimental conditions the NADH profile of *eno1*Δ mutant was very similar to the wild type (data not shown).

The conversion of phosphoenolpyruvate to pyruvate (the last step in glycolysis) is catalysed by two pyruvate kinases encoded by *PYK2* and *CDC19*, respectively. However, Cdc19p appears to be the main pyruvate kinase because the *cdc19*∆ mutant is not able to grow in the presence of glucose [38]. Furthermore *CDC19* and not *PYK2* is tightly regulated and sensitive to levels of fructose-1,6-bisphospate. In agreement with these data, we found (data not shown) that glycolytic oscillations were present in the *pyk2*Δ and were comparable to the wild type.

**Figure 4:**
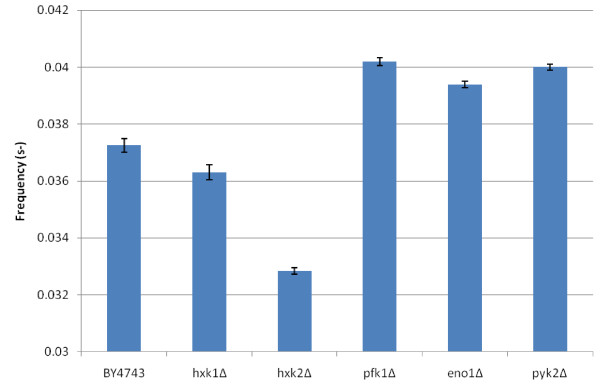
**Frequencies of glycolytic oscillations.** The frequencies of the oscillating parts of the NADH profiles for each mutant were found using Fourier analysis (Equation (1). The error bars are the standard deviation for three analytical replicates.

**Figure 5:**
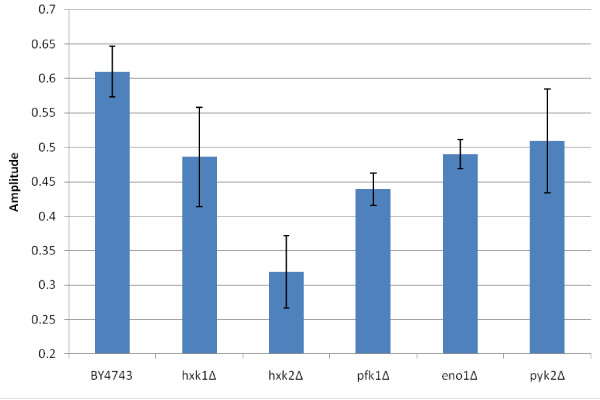
**Amplitudes of glycolytic oscillations.** The amplitudes of the oscillating parts of the NADH profiles for each mutant were found using Fourier analysis (Equation (1). The error bars are the standard deviation for three analytical replicates.

Fourier analysis was employed to determine the frequencies and amplitudes of the glycolytic oscillations in all mutants where the oscillations were present. The analysis shows that the frequencies (Figure 4) and amplitudes (Figure 5) of the mutants’ oscillations are largely within the error bars of the wild type BY4743 strain, with some exceptions. The most noticeable is the *hxk2*Δ mutant, whose frequencies and amplitudes are much lower. This can also be seen in the NADH profile (Figure 3a) of this mutant. Interestingly, this analysis shows that the mutants (*pfk1*Δ, *eno1*Δ and *pyk2*Δ) with an oscillatory pattern similar to that of the wild type have marginally higher frequencies and slightly lower amplitudes compared to the wild type. This suggests that these mutations also have a small effect on the oscillations, as opposed to no effect.

### Analysis of an existing model of glycolysis

The results described above provided a large amount of data that could on one hand be used to develop future models of yeast glycolytic oscillations, and on the other, to analyze published models. We have used the parameter sensitivity analysis tool in SBToolbox [26] as described in the Methods to analyze the sensitivities of the frequency and amplitude of NADH oscillations to model parameters using an existing model of glycolysis. Of the several published available models [18,20,22,30,39] we chose that of Wolf et al. [18] because it is comparatively easy to investigate and simulate (Table 2 and Figure 1); and because as shown by Brusch et al. [40], its dynamic properties are similar to those of the full scale glycolysis model [39].

**Figure 6:**
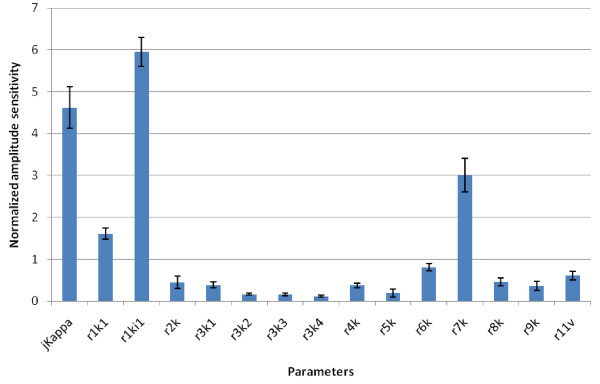
**Normalized amplitude sensitivities of the parameters of the Wolf model of glycolysis.** We have used the parameter sensitivity analysis tool in SBToolbox. The error bars are the standard deviation for the sensitivity of each species in respect to each parameter.

**Figure 7:**
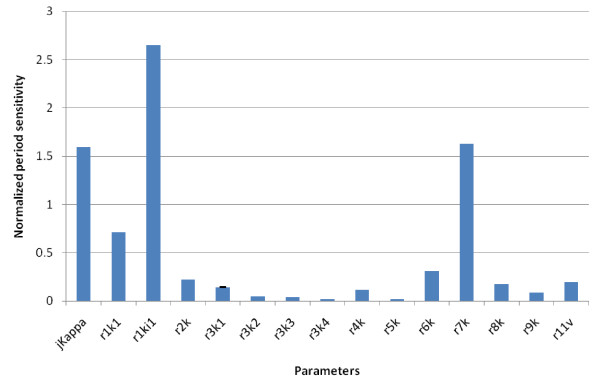
**Normalized period sensitivities of the Wolf model of glycolysis.** We have used the parameter sensitivity analysis tool in SBToolbox. The error bars are the standard deviation for the sensitivity of each species in respect to each parameter. Although error bars are plotted, the sensitivities of each variable were equal for each parameter so the errors are zero in all cases.

Based on our experimental evidence, we would expect the most sensitive parameters to be those of phosphofructokinase, and of the hexokinases (Figure 3b, a). The sensitivities of each model parameter in respect to amplitude (Figure 6) and periodicity (Figure 7) were analysed. Our data shows that the most sensitive parameter is *r1ki1*, the inhibition constant for the lumped HK-PFK reaction. Interestingly, *r7k*, the rate constant for the ATPase reaction of the model is another sensitive parameter. This suggests that ATPases have a key role in regulating glycolytic oscillations, a possible example of distributed control [22]. The next most sensitive parameter is *jKappa*, the rate constant for acetaldehyde diffusion, which is evidence for the importance of acetaldehyde in controlling the oscillations.

**Figure 8:**
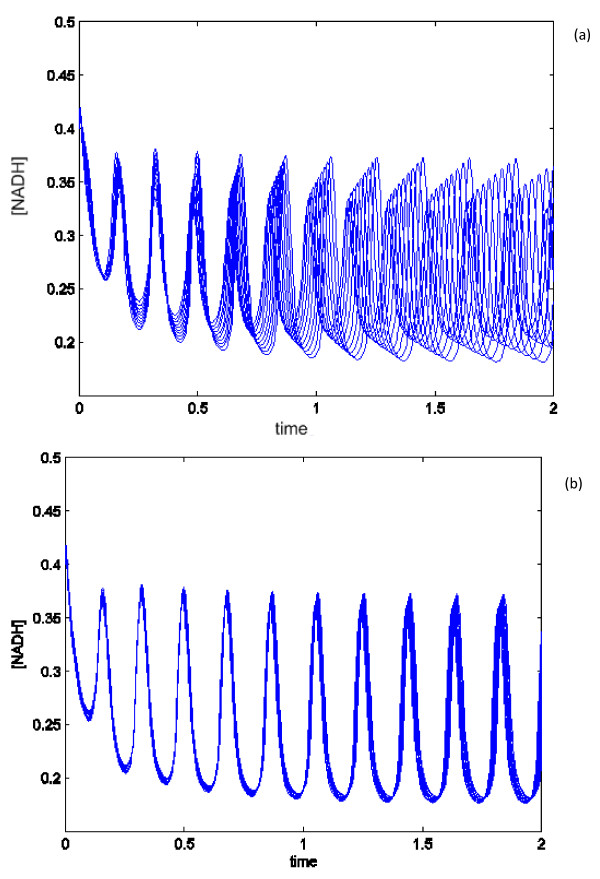
**Perturbation analysis of the Wolf model.** Panels: (**a**) perturbations of the most sensitive parameter *r1ki*1by applying 1% increments for up to 10% in total; (**b**): perturbations of one of the least sensitive parameter *r8k* by applying 1% increments for up to 10% in total.

In order to illustrate the effects of the sensitivity of some of the parameters identified in our analysis (Figures 6 and 7) we simulated the Wolf model varying *r1ki1* (the most sensitive parameter) or *r8k* (one of the least sensitive parameters) by applying 1% increments for up to 10%, and the results of the simulations are plotted. While there were clearly differences in frequency between the simulations upon perturbation of *r1ki1* (Figure 8a), there was little if any change in the NADH frequency in the simulations upon perturbation of *r8k*. This indicates that simulation of the Wolf model agrees with our experimental results (Figure 3) as deletion of *PFK2* and *HXK2* genes had the greatest effect on the oscillations.

**Figure 9:**
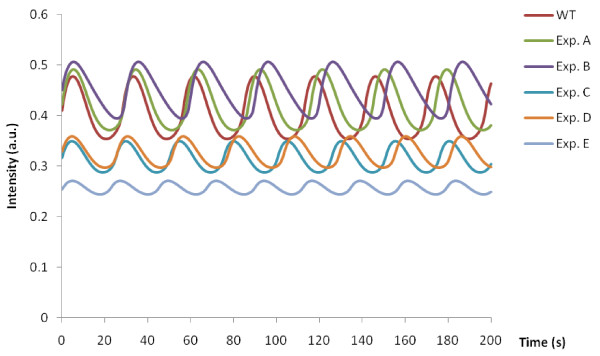
**Simulations of glycolytic oscillations as a result of changes to the GAPDH levels in the Wolf model of glycolysis.** (**A**) - 50% reverse; (**B**) – 0% reverse; (**C**) – 50% forward; (**D**) – 50% forward and reverse; (**E**) – 30% forward. Resulting effects on parameter values are shown in Table 3.

Our genetic analysis demonstrated a differential role for the glyceraldehyde-3-phosphate dehydrogenase isoforms: deletion of *TDH1* and *TDH2* had little effect on glycolytic oscillations (Figure 3c) while that of *TDH3* led to a loss of oscillations. It has been reported that Tdh2 and Tdh3 are both functional in exponentially growing cells. Importantly, expression of *TDH3* contributes 50-60% of normal glyceraldehyde-3-phosphate dehydrogenase activity [32] during exponential growth phase, which we hypothesised could help explain why deletion of *TDH3* and not that of *TDH2* affects oscillations. We used the Wolf glycolysis model to test this hypothesis, by simulating the effects of reducing GAPDH activity both in the forward or reverse reaction. The results of this analysis (Figure 9 and Table 3) showed that changes to levels of GAPDH activity affect glycolytic oscillations if incorporated in the forward but not the reverse reaction. Decreasing enzyme levels to even 0 in the reverse reaction had no effect on the amplitude and period of oscillations (Figure 9). A reduction of 50% in the forward reaction decreased significantly the amplitude and slightly the period, but oscillations were maintained. An additional 50% decrease in the reverse reaction made no discernable difference (Figure 9 and Table 3). In contrast, reducing GAPDH levels below 30% in the forward reaction abolished oscillations almost completely (Figure 9).

**Table 3:** Results of simulations of the Wolf model upon changing GAPDH levels

**Simulation set**	**Forward reaction**	**Reverse reaction**	**Amplitude**	**Period**
	**r3k1**	**r3k2**		
WT	323.8	57823.1	0.1239	28.600003
Exp A	100%	50%	0.1188	29.44121
Exp B	100%	0	0.1121	30.33336
Exp C	50%	100%	0.0614	25.02503
Exp D	50%	50%	0.0614	26.34213
Exp E	30%	100%	0.0271	20.02002

### Constitutive activation of the cAMP pathway abolishes glycolytic oscillations

**Figure 10:**
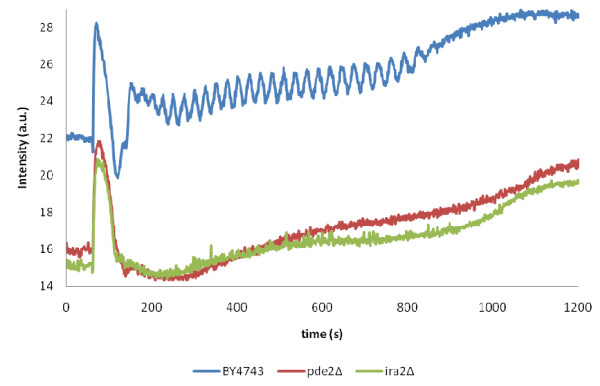
**Glycolytic oscillations in the wild type BY4743 compared to isogenic mutants in genes encoding respective components of the cAMP signal transduction pathway: BY4743 (blue trace);*pde2*Δ (red trace),*ira2*Δ (green trace).** Oscillations were induced by the addition of glucose to a concentration of 30 mM after 60 seconds, followed by addition of KCN to a final concentration of 5 mM after 140 seconds. NADH fluorescence intensity was followed using a fluorimeter.

Previously we have reported that deletion of the gene encoding the high affinity cAMP phosphodiesterase Pde2p causes significant changes to the transcript abundances of the majority of the genes encoding glycolytic enzymes [41-43]. Since Pde2p is a key component of the protein kinase A (PKA) signal transduction pathway – the key regulator of nutrient sensing, metabolism, and the diauxic shift in yeast, these results prompted us to investigate what effect this pathway had on glycolytic oscillations. Of the deletion mutations of genes encoding components of the pathway that we tested, we observed wild type patterns of glycolytic oscillations in *pde1*Δ (*PDE1* encodes the low affinity cAMP phosphodiesterase: [44]); *gpr1*Δ (*GPR1* encodes the G-protein coupled glucose receptor: [45]), *gpa2*Δ (*GPA2* encodes the alpha subunit of a heterotrimeric G protein: [46]), *tpk1*Δ, *tpk2*Δ and *tpk3*Δ mutants (*TPK1, TPK2* and *TPK3* encode the three isoforms of the catalytic subunit of PKA [47]) (data not shown). In contrast, the glycolytic oscillations were completely abolished in *ira2*Δ (*IRA2* encodes a GTPase activating protein- Ras-GAP, responsible for inactivating Ras-GTP [48]) and *pde2*Δ mutants (Figure 10). It is noteworthy that in both *ira2*Δ and *pde2*Δ mutants the cAMP-PKA pathway would be in a constitutively activated state [49]. Remarkably, out of the large number of interactions (physical and genetic) reported in the SGD data base (http://www.yeastgenome.org/), we noted that while both Pfk1 and Pfk2 have been shown to physically interact with PKA catalytic subunit isoforms (PFK is a known phosphorylation target for PKA), only *PFK2* has been found to interact genetically with *IRA2* and *PDE2,* whose deletion mutations abolish glycolytic oscillations.

## Discussion

This study reports the results of the first genetic investigation of the pattern of oscillations of intracellular NADH of single deletion mutants in key components of the glycolysis pathway in *S. cerevisiae*. It has become possible because of the genetic resources available when using the sequenced genetic background strain BY4743, which as our study shows (Figure 2) displays an NADH oscillatory pattern identical to that of strain X2180 used in previously reported experiments.

One of the most significant results of this study is the genetic evidence of the differential roles of the two subunits of phosphofructokinase (PFK) in glycolytic oscillations. Previously, mathematical modelling which has demonstrated that the phosphofructokinase, with its allosteric regulation, has the greatest control over the oscillations has not discriminated between the two types of subunits that constitute this enzyme. Lack of PFK activity *in vivo* is only observed in double *pfk1 pfk2* deletion mutants. In contrast, single deletion mutants (*pfk1* and *pfk2*) can grow on glucose, although there is no *in vitro* detectable PFK activity in these mutants [31]. Arvanitidis and Heinisch [50] demonstrated that each of the phosphofructokinase subunits can serve catalytic as well as regulatory functions. However, while the homomeric nature of the PFK enzyme supports growth on glucose, strains that have only one of the Pfk subunits have other different phenotypes. For example Rodicio et al. [51] have shown that only the *pfk2* deletion mutant is temperature sensitive for growth at 37°C, sensitive to caffeine and Congo red. The *pfk1* deletion mutant on the other hand is more resistant to caffeine compared to the wild type. And in the current study we show further phenotypic differences between the two types of deletion mutants. Firstly, the *pfk2* but not *pfk1* deletion mutant is deficient in NADH-mediated glycolytic oscillations. Secondly, *pfk2* and not *pfk1* mutant does not display the characteristic NADH “spike” after glucose addition (Figure 3b), an observation which will be the subject of further investigations.

The specific role of Pfk2 in glycolytic oscillations could be due to the fact that it is the subunit which contains binding sites for fructose 6-phosphate [52], as previous studies have shown that yeast extracts fed with the PFK substrate fructose 6-phosphate can exhibit oscillations, whereas extracts fed with fructose 1–6 bisphosphate cannot [29]. On the other hand, the differential role of the two subunits of PFK could also be due to the differences in their associations with the cAMP pathway, which as this study shows is responsible for modulating the enzyme activities of the glycolytic enzymes in such a way that the system becomes unstable and starts to oscillate. Unlike *PFK1*, *PFK2* is involved in genetic interactions with *PDE2* and *IRA2*, the components of the cAMP pathway which affect NADH oscillatory activity (Figure 10). Pde2 and Ira2 are both responsible for feedback down-regulation of the cAMP-mediated activity of PKA [49]; hence deletion in either encoding gene causes constitutive activation of the cAMP pathway. The exact mechanism by which the cAMP-PKA pathway affects glycolytic oscillations is currently unknown, but it could be hypothesized that it might be via phosphorylation of Pfk2 by PKA, as Pfk2 (but not Pfk1) has been shown to physically interact with Tpk1. However, the fact that the pattern of glycolytic oscillations of the *tpk1*Δ mutant is wild type suggests that Tpk1-mediated phosphorylation of Pfk2 does not contribute to its role in glycolytic oscillations. However, it cannot be ruled out that in the absence of Tpk1, another PKA isoform (Tpk2 or Tpk3) is capable of phosphorylating Pfk2.

Deletion of each of the hexokinases encoding genes leads to oscillations that are longer lasting with lower amplitude (Figure 3a), which agrees with earlier reports which have shown that reduced hexokinase activity results in lower flow of glucose into the glycolytic pathway [53]. Our study however, shows that the effect of *HXK2* deletion on the duration of the oscillations is stronger than that of *HXK1*. Although the deletion mutant for *GLK1* whose product is also known to phosphorylate glucose at the C6 position was not investigated, we predict that its deletion would yield results comparable to those of *HXK1*, as both *GLK1* and *HXK1* are repressed when grown on a fermentable medium using glucose as a carbon source [54]. Interestingly, the transcript abundances of both *HXK1* and *GLK1* have been reported to increase, whilst those of *HXK2* and *PFK2* decrease respectively, upon the diauxic shift [55], the transition known to be regulated by the cAMP signal transduction pathway. And remarkably, *HXK2* (but not *HXK1),* and *PFK2* (but not *PFK1*) have been found to interact genetically with *PDE2* and *IRA2*, deletions of which affect glycolytic oscillations (Figure 10). We therefore like to speculate that because of the role of the cAMP pathway in this oscillatory phenomenon, the glycolytic enzymes encoded by genes found to be genetically associated with *PDE2* and *IRA2* have the strongest regulatory role in glycolytic oscillations.

Deletion of *TDH1* and *TDH2* genes has little effect on glycolytic oscillations (Figure 3c). Deletion of *TDH3* however leads to a loss of oscillations. These isoforms are growth-phase specific: Tdh1 is present in stationary phase cells, while Tdh2p and Tdh3p are present in exponentially growing cells [33]. The observation that deletion of *TDH3* and not that of *TDH2*, affects oscillations could now be explained by the fact that expression of *TDH3* contributes around 60% of glyceraldehyde-3-phosphate dehydrogenase activity [32]. As demonstrated by our simulations of the GAPDH reaction in the Wolf model of glycolysis (Figure 9), glycolytic oscillations are maintained but affected in amplitude and period upon 50% reduction of the GAPDH activity in the forward reaction, and further decrease to 30% abolished them almost completely.

The question regarding effects of deletion of *ENO2* and *CDC19* (*PYK1*) in glycolytic oscillations remains as the corresponding mutants were not available to test in the current study. It could be hypothesised that their transcripts’ down-regulation upon the diauxic shift [55] suggests a role in glycolytic oscillations since a similar correlation exists for *PFK2*. And although there might be an enticing link between transcript down-regulation upon the diauxic shift of genes such as *HXK2* and *PFK2*[55] and their role in glycolytic oscillations, this relationship is not as straightforward. Our study shows that deletion of both *HXK1* and *HXK2* affects glycolytic oscillations, however their transcript levels respond in a contrasting manner to the diauxic transition: *HXK1* is up-regulated, whilst *HXK2* is down-regulated. Moreover, deletion of *TDH3* abolishes glycolytic oscillations completely although neither this gene, nor the remaining two (*TDH1,2*) are subject to differential regulation of transcription upon the diauxic shift.

Previously, Reijenga et al. [56] and Madsen et al. [30] reported the control coefficients of enzymatic steps on the amplitude and frequency of oscillations, providing a measure of the effect that changes in enzyme activity produce on these characteristics. The deletion mutations in the present investigation represent large perturbations of the glycolysis pathway components whereas parameter sensitivities refer to changes in individual reaction parameters. This type of analysis has enabled us to distinguish between forward and reverse reaction parameters in reversible reactions, and between rate and inhibition constants for the lumped HK-PFK reaction. It has identified *r1ki1,* the inhibition constant for the combined HK-PFK reaction, as the most sensitive parameter. Moreover, as our experimental evidence demonstrated it is the deletion of *PFK2*, *HXK1* and *HXK2* that produce the greatest effect on glycolytic oscillations. Deletion of *PFK1* on the other hand does not significantly affect glycolytic oscillations showing that the components of the lumped HK-PFK reaction have specific properties. More precise models will be needed that differentiate between the different isoforms or subunit components of glycolytic enzymes. Oscillatory parameter sensitivity analysis is a useful technique to identify crucial parameters for the control of biological rhythms and investigate their robustness. It has been applied to several other types of oscillating systems, such as circadian rhythms [28,57], NF-κB signalling pathways [58] and calcium dynamics [59].

Interestingly, the forward constant for the lumped HK-PFK reaction (*r1k1*) is not as sensitive as the inhibition constant. This suggests that the reverse feedback by ATP on PFK is potentially the most important controller of the oscillations which is in agreement with results obtained in yeast extract experiments by Madsen et al. [30]. So not only have we identified the role of the beta subunit (Pfk2) of phosphofructokinase in glycolytic oscillations (as deletion of *PFK2* abolishes them completely), we have confirmed that the negative feedback of Pfk2 by ATP is a key regulatory factor in glycolytic oscillations.

The choice of the glycolysis model that we used in the current analysis might seem debatable. In light of the report by Madsen et al. [30] who argued firstly that there are two types of oscillations, namely relaxation type observed in cell free extracts and harmonic (sinusoidal) oscillations observed in intact cells, and secondly that the Wolf model is not applicable for oscillations in intact cells. However, it appears that the type of oscillations is not strictly coupled to the whole cells or cell extracts, but rather to how far the system is removed from the Hopf bifurcation. Close to this point the oscillations are harmonic, independent of whether they are observed in cell extracts or whole cells. Further from this point, the behaviour can move away from harmonic oscillations. This is for instance shown in Nielsen et al. [60], where cell free extract oscillations move from harmonic to complex and relaxation type of oscillations dependent on the distance from the Hopf bifurcation point. Furthermore, P. Richard [61] reports that the shape of the glycolytic oscillations varies from sinusoidal to relaxation depending on the rate of glucose addition.

This study demonstrated that *r7k*, the rate constant for the ATP-ase reaction, is also a highly sensitive parameter (Figures 6 and 7). Recently, Kloster and Olsen [62] investigated the role of intracellular ATPase activity using specific inhibitors for the three types of ATPases, namely F_0_F_1_ ATPase, Pma1 and vacuolar ATPase, and showed that both inhibiting and stimulating ATPase activity suppress glycolytic oscillations. Further support of the role of ATPase in glycolytic oscillations comes from the recent study of Ytting et al. (2012) [63]. However, the latter study uses a *GAL1* promoter replacement *pma1* mutant and does not present data for its isogenic wild type parental strain. Instead the report shows as supplementary data the results of an alternative wild type strain (a derivative of W303) with a NADH oscillatory pattern which is very far from comparable to X2180 and that of BY4743 shown in our study. There are different ATP-ase encoding genes in the yeast genome; however none of the respective viable deletion mutants were included in our analysis. Given the well established role of the genetic background as a modifier of different phenotypes, we propose that in future studies it would be of interest to investigate the role of the different ATP-ases further by analysing the NADH-mediated oscillations in the standard BY4743 genetic background.

## Conclusions

This study has for the first time provided genetic evidence for the differential roles of the two types of subunits of PFK, and the isoforms of GAPDH and HK in glycolytic oscillation in the yeast *S. cerevisiae*. Importantly it shows genetic evidence that *PDE2* and *IRA2*, which encode components of the cAMP pathway responsible for negative feedback regulation of PKA, are required for glycolytic oscillations, suggesting an enticing link between these cAMP pathway components and the glycolysis pathway enzymes with a key role in glycolytic oscillation. We demonstrate that a systematic genetic approach combined with mathematical modelling can advance the study of oscillatory phenomena.

## Competing interests

The authors declare no competing interests in relation to this manuscript.

## Authors' contributions

TW carried out the characterization of the NADH-mediated glycolytic oscillations profiles of the mutants and the initial Wolf’s model simulation. He was also involved with the drafting of the manuscript, and preparation of figures and tables. DA performed the normalized parameter sensitivity analysis, simulated the effects of reducing GAPDH activity, and helped JMS in preparing Figure 9 and Table 3. JMS provided guidance and help with the model simulations and parameter sensitivity analysis, constructed Figure 1, and helped critically revise the manuscript, the figures and the figure legends. LS conceived of the study, and was instrumental in the experimental design and coordination, as well as drafting and revising the manuscript. All authors read and approved of the final manuscript.
